# Testing a vigorous intermittent lifestyle physical activity intervention in adults transitioning to retirement: a pilot randomised controlled trial

**DOI:** 10.1093/ageing/afaf244

**Published:** 2025-09-16

**Authors:** Bingyan Pang, Joanna C Moullin, Craig Thompson, Cecilie Thøgersen-Ntoumani, Emmanuel Stamatakis, Matthew Ahmadi, Joanne A McVeigh

**Affiliations:** School of Allied Health, Curtin University, Perth, Western Australia 6845, Australia; School of Allied Health, Curtin University, Perth, Western Australia 6845, Australia; enAble Institute, Bentley, Western Australia 6102, Australia; School of Allied Health, Curtin University, Perth, Western Australia 6845, Australia; enAble Institute, Bentley, Western Australia 6102, Australia; School of Allied Health, Curtin University, Perth, Western Australia 6845, Australia; University of Southern Denmark, Odense 5230, Denmark; University of Sydney, Sydney, New South Wales, Australia; University of Sydney, Sydney, New South Wales, Australia; School of Allied Health, Curtin University, Perth, Western Australia 6845, Australia; enAble Institute, Bentley, Western Australia 6102, Australia; University of Witwatersrand, Braamfontein Johannesburg 2017, South Africa

**Keywords:** ageing adults, behaviour change, vigorous intermittent lifestyle physical activity, older people

## Abstract

**Background:**

Vigorous intermittent lifestyle physical activity (VILPA; short bursts of vigorous-intensity activities in a person’s daily life) could be an attractive and feasible option to increase physical activity (PA) in adults transitioning to retirement.

**Design and setting:**

Two-arm pilot randomised controlled trial (RCT) to test the feasibility of the intervention and the plausibility of the intervention to increase PA in adults transitioning to retirement in Perth, Western Australia.

**Participants:**

Insufficiently physically active adults transitioning to retirement.

**Intervention:**

Twelve-week theory-based and evidence-informed VILPA intervention designed to increase PA in adults transitioning to retirement.

**Objectives and measurements:**

The feasibility of the pilot was determined by the projected sample size with actual sample size, drop-out rates and reporting rates. The feasibility, acceptability and appropriateness of the intervention were assessed using validated questionnaires. The intervention’s plausibility to increase PA was assessed by accelerometer-measured PA, functional fitness test and general health questionnaire.

**Results:**

Eighty individuals expressed interest in participating in the trial; 42 (feasibility of recruitment = 52.5%) were recruited and 34 completed the trial (retention = 80%). The preliminary data indicated increases in both total PA and VILPA, with positive impacts in self-reported general health and functional fitness. Participants found the intervention acceptable and intended to continue participation in VILPA and accumulate PA after the intervention.

**Conclusions:**

The VILPA intervention appears to be feasible for promoting PA in ageing adults. The findings of this pilot RCT also support a larger trial to seek the effectiveness of VILPA in improving health outcomes in ageing adults.

## Key points

- Vigorous intermittent lifestyle physical activity could be a feasible option to encourage physical activity in ageing adults.
- The VILPA intervention demonstrated potential to increase physical activity in adults transitioning to retirement.
- Adults transitioning to retirement found the VILPA intervention feasible and acceptable.

## Background

Promoting physical activity (PA) in the ageing population is a global public health priority [1]. One in three adults worldwide is insufficiently physically active (i.e. not meeting the World Health Organisation’s recommendations accumulating 150 to 300 min of moderate-intensity PA or 75 to 150 min of vigorous-intensity PA per week), with physical inactivity contributing to $53.8 billion per year in healthcare costs [1, 2]. The prevalence of insufficiently physically active adults increases with age and adults aged ≥55 years are the least active [1, 3]. In 2024, the World Health Organisation reported adults are off track to meet PA recommendations globally [1]. The commonly reported barriers to participation in traditional exercises among older adults are ill health, fear of injury, lack of access to facilities and cost [4]. More attractive and feasible options for the ageing population are needed to encourage PA in their daily life.

One potential strategy to encourage PA is through short bouts (<1–2 min [5]) of vigorous intermittent lifestyle physical activity (VILPA; e.g. climbing a few flights of stairs, carrying a small load of shopping) [6]. Non-exercising adults (mean age of 62 years) who engaged in VILPA bouts <1–2 min per bout, three times per day, were shown to have ~40% lower all-cause mortality than those who did not engage in VILPA. Participation in a total of ~4 min of VILPA per day was also associated with an 18% reduction in total incident cancer risk among non-exercising adults [7]. VILPA may be a more attractive and feasible alternative for ageing adults who do not wish or are not able to participate in traditional exercise programs [8].

To date, there are a handful of interventions that encourage VILPA-like activities and/or small bouts of exercise in the ageing population [9]. However, these interventions tend to focus on short bursts of exercises outside of a person’s daily routine. Using a published four-pillar VILPA research framework [6], we developed an intervention guided by the Behaviour Change Wheel methodology [10]. The key stages of the VILPA intervention development involved (i) a literature review [11] of existing evidence on barriers and facilitators to participation in vigorous lifestyle PA in ageing adults, (ii) mapping the barriers and facilitators to behaviour change theory frameworks, (iii) seeking the target population’s perspectives on VILPA [8], (iv) identifying intervention options [8], (v) consulting with stakeholders to adapt the contents of the intervention, and (vi) determining implementation strategies and the modes of delivery for the support materials. The final VILPA intervention is theory based and evidence informed, targeting adults transitioning to retirement (i.e. recently retired in the past 6 months or planning to retire in the next 5 years). The transition to retirement was chosen as, during this period, ageing adults face many life changes, such as income, support networks, the likelihood of a diagnosis of non-communicable diseases, and lifestyle [12]. Life transitions disrupt existing cues and routines and create a temporary state where individuals are more receptive to forming new habits. This temporary state offers a possibility to identify new cues/triggers that can elicit a new desired PA behaviour. As such, the period when someone transitions to retirement presents a unique window of opportunity to intervene [12, 13].

The overarching aim of this study was to pilot the developed intervention designed to increase participation in VILPA and overall PA in adults transitioning to retirement and determine the feasibility of the intervention before initiating a large-scale trial to investigate its efficacy. The specific objectives were to examine (1) the feasibility of recruitment and completion of the intervention, (2) the appropriateness of the selected measures to examine and report effects on the intervention, (3) the acceptability of the intervention and (4) the plausibility of the intervention in increasing PA.

## Method

### Study design

This study was a two-arm pilot randomised controlled trial (RCT) that ran in the Perth metropolitan area of Western Australia in 2023. Eligible participants were randomised to either the intervention or waitlist-control group. The study sample size followed established guidelines for pilot RCTs [14].

### Ethics

Curtin University Human Ethics Research Committee approved this study HRE2022-0304. This pilot study was registered with the Australian and New Zealand Clinical Trial Registry, trial ID ACTRN-12623000493640. All participants provided informed consent in this study.

### Participants

This study aimed to recruit 80 participants from June to August 2023. Participants were recruited through social media, local newspapers, radio station adverts and organisation emails. The inclusion criteria were (i) adults who self-identified as recently retired in the past 6 months or planning to retire in the next 5 years, (ii) have no life-threatening physical or mental health conditions that prevent them from doing VILPA, (iii) speak English, (iv) lives in Perth metropolitan area, (iv) willing to attend Curtin University for all measurements and (v) not currently engaging in regular exercises designed to improve or maintain physical fitness. Participants were excluded if they were routinely engaging in a total of 10 min or more of PA, enough to raise their breathing rate, for >3 days a week. Each prospective participant was screened with the inclusion criteria by the first author (Appendix 1) and sought family doctor’s advice before enrolling in the study (Appendix 2).

### Randomisation

Eligible participants were randomised by a computer-generated block size of five by the first author. The number of participants in each arm aimed to achieve a one-to-one ratio (40 in each arm). The randomisation method is outlined in the CONSORT 2010 statement [15].

### Intervention and study procedures

**Figure 1:**
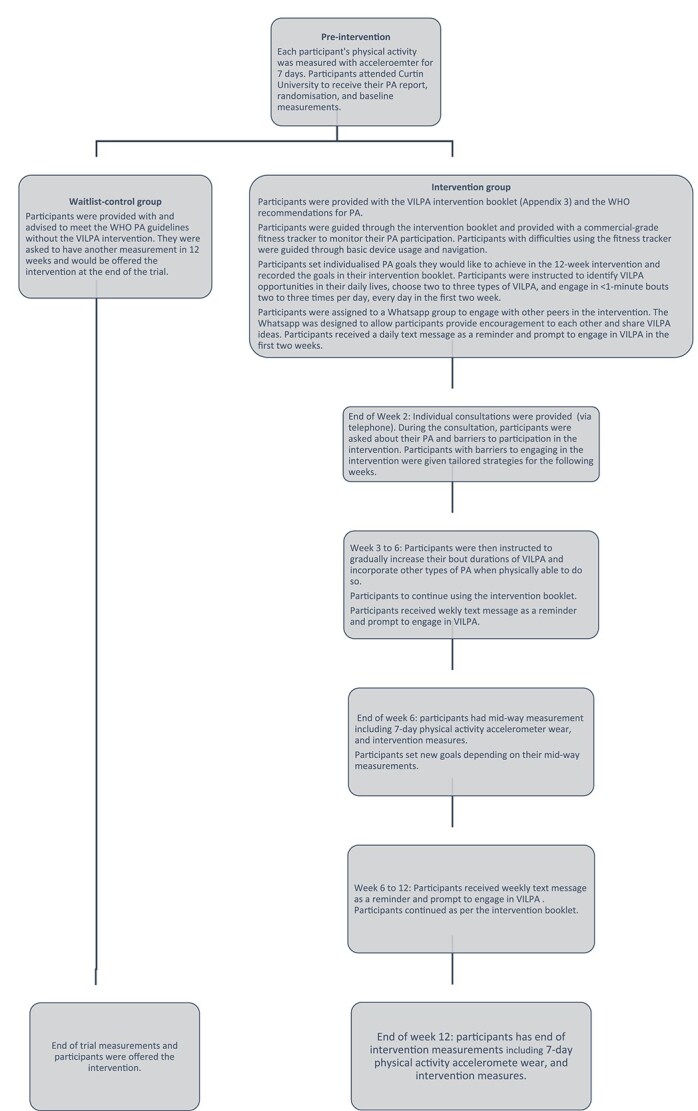
Intervention and study procedures.

In brief, the intervention consists of the increase of awareness of PA recommendations and health benefits associated with vigorous-intensity PA (VPA; enough to increase their breathing rate) in older age, the identification of individuals’ vigorous intensity and VILPA opportunities, goal setting, weekly checklists, regular reminders, self-monitoring of PA, rewards and social support. The intervention and study procedures are outlined in Fig. 1. Details of the intervention are provided in Appendix 3. Intervention was provided by BP who is an allied health clinician with 11 years of clinical experience in working with people with chronic health conditions.

### Data collection

Sociodemographic information was collected upon each participant’s enrolment into the study (see Appendix 4 for details). Physical activity and health outcomes measures were collected at baseline, 6 weeks and at the end of the intervention (12 weeks) of the intervention group and at two timepoints (baseline and the end of 12 weeks) of the waitlist-control group.

### Measures

#### Feasibility, appropriateness and acceptability

Feasibility of the pilot RCT was assessed by examining the projected sample size with actual sample size, drop-out rates and reporting rates. Feasibility of the intervention was assessed by a rating scale [16, 17] from the participants’ perspective. Participants’ perceived appropriateness and acceptability of the intervention were assessed using surveys adapted to this study at the end of the intervention [16].

#### Habit formation

Habit formation was measured by the self-reported habit index [18] and the 16-item situational motivation scale [19]. They were adapted to assess VILPA habits and motivation at the end of the intervention. Participants’ intention to continue participation in VILPA was assessed by the Measure of Intention [17].

The health outcome measures (detailed in Appendix 4) and PA included the following:

- General health and wellbeing measured by the 36-item Short Form Health Survey (SF-36; https://www.rand.org/) [20, 21].
- Heart rate and blood pressure were measured using an automated blood pressure monitor.
- Functional fitness was assessed with the 6MWT [22].
- Physical activity was measured with an accelerometer (Actigraph GT9X; programmed to record raw data at 30 Hz) worn on the participant’s dominant wrist for seven continuous full days. Raw accelerometery data were exported using ActiLife version 6.13.5. The accelerometer-measured data were processed in two ways. For individual PA feedback and increasing awareness of PA intensities and accumulation, a clinical report (60-s epoch and cut points by Montoye *et al*. [23]) was generated for each participant. For the purpose of evaluating the plausibility of the intervention to encourage PA, raw accelerometer data were processed using the validated accelerometer-based two-level Random Forest machine learning activity scheme [24–27]. The two-level Random Forest machine learning–based schema development, validation and statistical analysis procedures have been published elsewhere [24–27].

### Data analysis

Statistical analysis included descriptive statistics and percentages for each group. Descriptive statistics were compared between intervention and waitlist-control groups. No statistical tests were performed to examine the effects of the intervention in this study due to its pilot-trial nature [28, 29].

## Results

### *Sample characteristics* (see Table 1)

**Table 1:** Sample characteristics and health measurements

	Int^Baseline^ (*n* = 21)	Int^Midway^ (*n* = 15)^a^	Int^End^ (*n* = 17)	Wait^Baseline^ (*n* = 20)	Wait^End^ (*n* = 17)	Total^Baseline^ (*n* = 41)	Total^End^ (*n* = 34)
**Age—years, mean (SD)**	65.3 (4.1)			66.3 (5.3)		66.0 (4.7)	
**Gender (*n*)**							
Male	6	4	4	8	7	14	11
Female	15	11	13	12	10	27	23
**Retirement status (*n*)**							
Retired	2	2	2	4	4	6	6
Working	19	13	15	16	13	35	28
**Working type prior to retirement (or current) (*n*)**							
Manual	4			4		8	
Non-manual	17			16		31	
**Education**							
Postgrad	3			4		7	
Bachelor	7			7		14	
Diploma	4			3		7	
Certificates/high school	7			6		13	
Number of chronic health conditions	1.8 (range = 0 to 6)			2.7 (range = 0 to 9)		2.2 (range = 0 to 9)	
**Health measurements mean (SD)**	Int^Baseline^ (*n* = 21)	Int^Midway^ (*n* = 15)^a^	Int^End^ (*n* = 17)	Wait^Baseline^ (*n* = 19)^b^	Wait^End^ (*n* = 17)	Total^Baseline^ (*n* = 41)	Total^End^ (*n* = 34)
Resting heart rate (BPM)	73 (10)	66 (8.2)	67 (11)	69 (8.3)	74 (9.9)	71 (9.4)	71 (11)
Resting systolic BP (mmHg)	135 (11.5)	126 (11.2)	128 (15)	126 (14.2)	122 (13)	131 (13.2)	125 (14)
Resting diastolic BP (mmHg)	84 (8.2)	82 (6.6)	79 (9.5)	77 (7.8)	75 (10)	81 (9.0)	77 (9.8)
6MWT in metres	579 (79.4) (range = 390 to 700)	656 (76.6) (range = 460 to 770)	657 (93) (range = 445 to 790)	596 (68.5) (range = 430 to 700)	609 (76.6) (range = 430 to 720)	587 (74) (range = 390 to 700)	633 (87) (range = 430 to 790)
**SF-36 mean (SD)**	Int^Baseline^ (*n* = 21)	Int^Midway^ (*n* = 15)^a^	Int^End^ (*n* = 17)	Wait^Baseline^ (*n* = 20)	Wait^Post^ (*n* = 17)	Total^Baseline^ (*n* = 41)	Total^End^ (*n* = 34)
Physical functioning	84 (14.3)	84 (13.1)	91 (9.2)	83 (16.4)	88 (12)	83 (15)	89 (10)
Role limitation due to physical functioning	85 (30.1)	75 (30.6)	81 (37)	81 (32.3)	75 (29)	83 (31)	78 (36)
Role limitation due to emotional wellbeing	79 (35.8)	70 (35.1)	84 (31)	87 (29.4)	88 (29)	83 (33)	86 (30)
Energy	56 (15.7)	63 (14.1)	67 (19)	60 (20.4)	61 (22)	58 (18)	64 (20)
Emotional wellbeing	77 (15.6)	76 (17.6)	84 (14)	78 (13.3)	78 (15)	78 (14)	81 (15)
Social function	82 (24.5)	84 (22.3)	89 (18)	85 (20.5)	88 (18)	84 (22)	88 (17)
Pain	76 (22.3)	83 (18.6)	78 (18)	77 (21.0)	74 (19)	77 (21)	76 (18)
General health	66 (15.4)	72 (14.6)	78 (20)	65 (18.4)	68 (16)	65 (17)	72 (19)

Note: The SF-36 survey findings are converted to scores out of 100 in each domain. The higher the value, the better the outcome.

Abbreviations: BP = blood pressure. BPM = beats per minute. Int = Intervention group. Wait = waitlist-control group. SD = standard deviation. Midway = week 6 measurement in the intervention group. Post = end of 12-week trial measurement.

^a^Three participants withdrew from the intervention before mid-way measurement, three participants were unwell and could not attend mid-way measurement.

^b^One participant did not have 6MWT measured due to the accidental discovery of hypertension. The participant was advised to seek further medical clearance.

The participants’ mean age was 65.0 years in the intervention group and 66.3 years in the waitlist-control group. The ratio of males to females, retirement status, work type, education and chronic health conditions were similar in both groups.

### Feasibility

**Figure 2:**
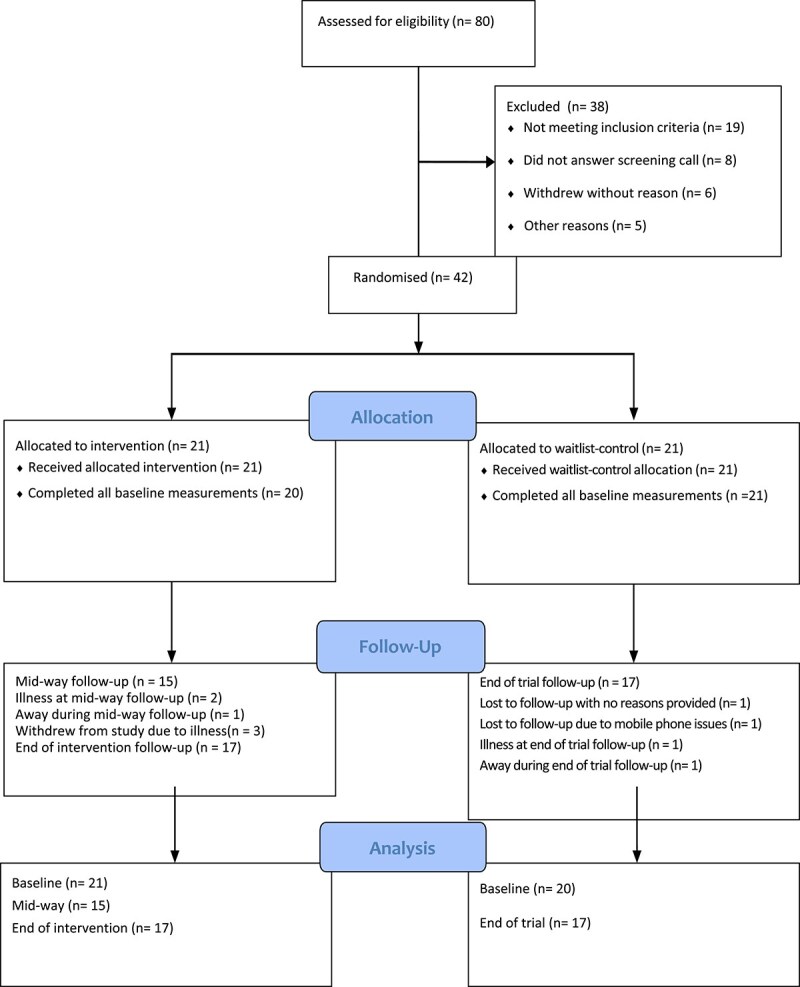
CONSORT flow chart.

Eighty people who expressed interest in the study were assessed for their eligibility. Forty-two people (52.5% recruitment target) met the selection criteria and consented to participate (see Fig. 2 CONSORT flow chart). Out of the 42 enrolled participants, 34 (81%) completed the 12-week trial. All participants (*n* = 42) were willing to be randomised. A one-to-one ratio was achieved with 21 participants in each arm. All 42 participants completed pre-trial accelerometer measurements, and 41 participants (97.6%) completed pre-trial general health and 6MWT measurements. One participant was lost to follow-up due to mobile phone issues. Forty participants (95%) completed pre-trial 6MWT.

In the intervention group, 15 participants (71.4%) completed mid-way measurements; and 17 participants (81.0%) in the intervention group completed final measurements. In the waitlist-control group, 17 participants (81.0%) completed final measurements.

All 17 participants who completed the intervention reported the intervention was feasible, appropriate and acceptable (see Table 2).

**Table 2:** Feasibility, appropriateness and acceptability survey results and summary findings of measurement of intention at week 12 of the intervention (*n* = 17)

No.	Contents	Mean (SD)
I. Feasibility of intervention measure		
1	The program was implementable.	4.65 (0.49)
2	The program was possible.	4.71 (0.47)
3	The program was doable.	4.65 (0.49)
4	The program was easy to use.	4.76 (0.44)
II. Appropriateness of the program measures		
1	The program seemed fitting.	4.53 (0.51)
2	The program seemed suitable.	4.59 (0.51)
3	The program seemed applicable.	4.53 (0.51)
4	The program seemed like a good match.	4.59 (0.51)
III. Acceptability of the program		
1	The program met my approval.	4.59 (0.62)
2	The program was appealing to me.	4.76 (0.44)
3	I liked the program.	4.82 (0.39)
4	I welcomed the program.	4.88 (0.33)
IV. Measurement of intention		
1	I plan to do VILPA after the program.	4.29 (0.69)
2	Continuing doing VILPA is high priority to me.	4.41 (0.62)
3	I will continue to do all aspects of this program.	4.24 (0.75)

Note: For measures of feasibility, appropriateness and acceptability, the Likert scale for each item was converted to a score: 1 = strongly disagree, 2 = disagree, 3 = neither agree or disagree, 4 = agree, 5 = strongly agree. The mean of each response was calculated [16]. For measurement of intention, the Likert scale for each item was converted to a score: 1 = not at all, 2 = to a slight extent, 3 = to a moderate extent, 4 = to a great extent, 5 = to a very great extent.

### Physical activity

At baseline, 21 intervention group participants and 20 waitlist-control group participants had accelerometer measurements. The baseline mean valid number of days of accelerometer-measured PA of both groups was 6.3 days (intervention group = 6.5 days; waitlist-control group = 6.1  days). As shown in Table 3, the baseline mean duration of VPA per day of participants was similar in both groups. The mean duration of VPA and VILPA bouts per day of participants in the intervention group increased at the mid-way and end-of-trial measurements.

**Table 3:** The mean duration of vigorous-intensity physical activity per day in minutes and mean VILPA bouts per day

	Intervention group			Waitlist-control group	
	Baseline	Midway	Post-trial^a^	Baseline	Post-trial
VPA per day in minutes	2.2 (2.9) (range: 0.1–10.7)	6.0 (4.4) (range: 0.5–14.6)	5.8 (7.1) (range: 0.1–28.2)	1.9 (2.0) (range: 0.0–7.3)	2.8 (3.7) (range: 0.0–11.6)
VILPA bouts	6.5 (6.2) (range: 0.7–21.9)	11.2 (7.3) (range: 2.4–26.3)	8.5 (6.8) (range: 0.4–21.4)	6.6 (6.6) (range: 0.1–20.5)	5.5 (5.6) (range: 0.2–21.9)

Abbreviations: VILPA, vigorous intermittent lifestyle physical activity; VPA, vigorous-intensity physical activity.

^a^For the end-of-trial measurement, one accelerometer from the intervention group was lost in the return mail. The results of the accelerometer-measured PA were therefore based on 16 participants.

The preliminary intervention effects on health outcomes and functional fitness are shown in Table 1. Decreased mean resting heart rates and blood pressures, and increased 6MWT distance at mid-way and end-of-intervention measurements were observed in participants in the intervention group compared to baseline measurements. Overall, participants in the intervention group reported better health and wellbeing in the 12-week intervention period.

### Habit formation

At the end of the intervention, participants reported that engaging in VILPA was becoming a part of their daily/weekly routine and that they intended to continue to do VILPA and other forms of PA (see Table 2 and Appendix 5).

## Discussion

This pilot two-arm RCT aimed to determine the feasibility of delivering a 12-week VILPA intervention, evaluate its preliminary effects in increasing PA in adults transitioning to retirement and provide foundational data for a full-scale RCT. This pilot RCT showed that the intervention is feasible and can increase VPA and participation in VILPA in the target population. Participants who completed the 12-week intervention found the intervention was acceptable and appropriate and intended to continue participation in VILPA and accumulate PA after the intervention. The pilot data also indicated that the intervention positively impacted individuals’ self-reported health and functional fitness.

The feasibility of recruitment (52.5%) and retention (80%) rates of this study are comparable to previous pilot studies on PA interventions in similar target populations [30–38]. Pilot studies targeting PA in ageing adults typically recruited 50 to 70 participants and retained 68% to 100% of the sample. Pilot studies with PA interventions targeting a specific clinical group, e.g. pre-frail [39], high falls risk [40] and post-stroke [41] adults aged ≥65 years, also reported similar recruited sample size and retention rates. Using a combination of recruitment strategies concurrently including self-enrolments and recommendations from key personnel (health professionals and peer mentors) could improve the recruitment and adoption rate in future studies.

Participants reported that the VILPA intervention was feasible, appropriate and acceptable. The positive results could be a result of the theory-informed and evidence-based multistage co-design process of the intervention which involved the target population and health professionals with experience in promoting PA [8, 42, 43]. This could also be a result of the simplicity of the intervention requiring individuals to simply increase the vigour of activities that already exist in their daily lives. With the increase in the vigour of the activities, participants also saw an improvement in their health outcomes, which was likely to have encouraged ongoing PA behaviour change. Participants intended to continue participation in VILPA after the intervention. This positive result could relate to the intervention requiring no special equipment (which differs from traditional exercise-based interventions), and participants are likely to maintain their VILPA levels post-intervention.

Pilot studies have a wide range of measures depending on the aim of the specific intervention [30–38]. In this pilot RCT, the selected measures were appropriate to compare preliminary intervention effects. The increases in the accelerometer-measured VPA and VILPA bouts were observed in the intervention group, corresponding to the increases in distance achieved in the 6MWT and better self-reported general health and wellbeing. Similarly, two previous pilot RCTs aimed to increase PA in ageing adults also showed increases in accelerometer-measured PA, and better self-reported general health and wellbeing [30, 31]. Other pilot studies of similar target populations tended to focus and report on feasibility measures and did not report on changes in PA participation [32, 33, 35, 38].

Increases in total distance achieved in 6MWT and VPA participation were found in participants in the intervention group at the mid-way measurement. These effects are likely to be attributed to the increased awareness of the benefits of short bursts of VPA (a key element of the intervention). Older adults with higher levels of knowledge of the benefits of PA are more likely to be active [44–46]. Participants’ engagement in more PA may have been positively affected by the education provided in the intervention, increasing their knowledge and perceived potential health-enhancing effects of doing lifestyle PA with more vigour. These improvements in the intervention group were maintained at the end-of-intervention measurement, resulting in greater VPA accumulation and 6MWT distance compared to the waitlist-control group. These improvements could be associated with the measurement and feedback of the participant’s individual PA at the mid-way of the program, which reassured and encouraged them to continue participating in VILPA and other forms of PA. These strategies in the intervention align with clinical recommendations [46, 47] that older adults are likely to face complex health challenges and should be regularly followed up when promoting PA. In addition, the 6MWT could be a low-cost and simple way to monitor and provide feedback on an individual’s PA participation. It is also more time-efficient and can be integrated into clinical settings compared to measuring PA using accelerometers. The improvements in functional fitness and general health of participants in the intervention group align with other interventions involving high-intensity interval training [48]. These findings suggest that some health changes achieved through traditional exercises could be achieved via daily activities.

The intervention effects of improved mean daily accumulation of VPA and self-reported general health and wellbeing appear to be greater than those of similar target populations in previous studies. A study aimed to increase PA in ageing adults by peer-led walking groups showed an increase of 1.1 min in daily VPA participation in 12 weeks; minimal changes were observed in participants’ self-reported general health outcomes [30]. Another study that tested an intervention to increase everyday activities in insufficiently physically active retiring women showed participation in 34 min of daily MVPA at 3 months (an increase of 10 min from baseline) [31]. However, no improvement was observed in their participants’ self-report general health. The VILPA intervention focused on performing activities that already exist in daily life at a vigorous intensity, which differs from interventions that require planning, time and location to perform and could be more convenient than structured exercise programs.

The findings of this study suggest that the intervention may have some potential to facilitate long-term PA behaviour change. Participants reported that engaging in VILPA was becoming a part of their daily routine and that they intend to continue to engage in VILPA after the intervention. This planned behaviour change could be a result of the intervention being implemented at a stage of life transition, focusing on meaningful activities that already occur in their daily lives and incorporating gradual PA intensity increases. The improvements observed in 6MWT distance and daily VILPA bouts suggest the intervention is likely to improve muscle strengthening and physical functioning. These improvements in physical functioning could be a result of engaging in daily tasks that are meaningful for the participants and thus maintaining ongoing engagement. Hence, with improvements in motivation and ongoing intention to engage in VILPA, and improved physical functioning, this intervention has the potential to facilitate long-term PA behaviour change.

### Limitations

The findings of this study should be interpreted with the following limitations in mind. First, the recruitment of this study required participants to self-enrol in the trial. Participants were interested in learning new strategies to be physically active. Individuals who were insufficiently physically active and less interested or motivated to engage in PA were not reached using the current recruitment strategy. Other studies should consider recruitment through various healthcare settings and form referral pathways to recommend insufficiently physically active individuals to enrol to the intervention.

Second, due to the nature of this study being a pilot trial, it was not powered to detect significant effects on PA and health outcomes. Therefore, the positive changes in PA and self-reported health outcomes should be interpreted with caution. Participants’ PA were measured by accelerometers on their wrists, which may have artificially inflated their participation in PA during mid-way and end-of-intervention measurements.

Nevertheless, the combination of the intervention delivered via a printed booklet, text message reminders and commercial-grade fitness trackers used in this study presented a relatively low-cost way to encourage participation in PA in ageing adults. The findings of this study also support the possibility of offering VILPA as an option in ageing adults to increase PA participation.

### Considerations and future recommendations

Communication between the intervention facilitator and participants likely encouraged PA participation. The facilitator of the VILPA intervention conducted intervention onboarding, measurements and follow-ups. The multiple follow-ups with participants built trust and rapport and may also have acted as reminders, accountability increasing and knowledge-building implementation strategies. The facilitators also guided participants in initiating a plan to identify VILPA opportunities in their daily lives.

Participants needed reminders to perform daily activities with more vigour. They also required assistance setting up the consumer-grade fitness trackers (~20 min for each participant). However, they reported positive experiences in self-monitoring their PA participation with the fitness trackers.

Participants were assigned to a WhatsApp group designed to provide social support and encouragement to each other. However, there were limited interactions among participants in this pilot trial. Future research could investigate more suitable modes to encourage social support and interactions between participants. The findings of this pilot RCT support the development of a larger trial seeking to examine the efficacy of VILPA interventions in improving health-enhancing PA, physical health and physical functioning in ageing adults. Future research could consider the implementation of the intervention in community settings and investigate the type and content of resources needed for health professionals to effectively promote VILPA.

## Conclusion

This pilot RCT demonstrated the feasibility of implementing a novel 12-week VILPA intervention for adults transitioning to retirement. The intervention was found to be appropriate and acceptable to the target population. The findings suggest the possibility of the intervention in increasing overall PA and vigorous activity participation in adults transitioning to retirement. However, this needs to be tested rigorously in a larger trial. Future research could investigate the health outcomes associated with participation in this VILPA intervention and strategies to optimise the dissemination and implementation of the intervention.

## Supplementary Material

aa_25_0581_File002_afaf244
