# Supplementary material for: Testing a vigorous intermittent lifestyle physical activity intervention in adults transitioning to retirement: a pilot randomised controlled trial
Source: Age Ageing. 2025 Sep 16;54(9):afaf244. doi: 10.1093/ageing/afaf244 (PMC12448735; doi:10.1093/ageing/afaf244)
Supplement: aa_25_0581_File002_afaf244 [file aa_25_0581_file002_afaf244.docx]

**Contents list**

Appendix 1: Participant eligibility screening tool

Appendix 2: Physical activity readiness and family doctor clearance form

Appendix 3: VILPA intervention booklet

Appendix 4: Data collection and health outcome measures

Appendix 5: Summary findings of self-reported habit formation and the situational motivational scale at the end of the 12-week intervention.

**Appendix 1: Participant eligibility screening tool**

Eligibility questionnaire for community participants – adults transitioning to retirement

Participant Name:

Question 1: Have you recently retired in the past 6 months?

A: Yes

B: No, I have fully retired more than 6 months ago. [Not eligible]

C. No, I am still working.

Question 2: If you are still working, are you planning to retire in the next 5 years?

A: Yes

B: No [Not eligible]

Question 3: Do you currently participate in any regular exercise (either on your own or in a formal class) designed to improve or maintain your physical fitness?

A: Yes [Not eligible]

B: No

Question 4: In the past week, on how many days have you done a total of 10 minutes or more of physical activity, which was enough to raise your breathing rate?

This may include sport, exercise, or brisk walking, or cycling, but should NOT include housework or activities that may be a part of your job.

A: More than 3 days [Not eligible]

B: 3 or less days

Question 5: Are you willing to wear a small device on your thigh for 7 days for measurement of your general health and physical activity?

A: No [Not eligible]

B: Yes

Question 6: Are you willing and able to attend at least two group discussions in person at ANONYMISED University to assist in developing a physical activity intervention?

A: Yes

B: No [Not eligible]

**End of survey.**

**Appendix 2: Physical activity readiness and family doctor clearance form**

Dear Doctor,

**Curtin University Human Research Ethics Committee (HREC) has approved this study (HRE2022-0304).**

Your client is interested in participating in a 12-week physical activity program and requires medical clearance to enrol. The program was developed with evidence from international literature and co-designed with health professionals and adults transitioning to retirement.

The 12-week program involves performing *Vigorous Intermittent Lifestyle Physical Activity (VILPA)* every day. The activities that can be done anywhere, anytime, do not need special equipment or skills to perform, and have the following unique characteristics:

They are high intensity – the persons doing the activity would have an increased breathing rate.

The activity only lasts a few minutes each time.

The activities are all daily living activities that already exist in a person’s life such as:

Brisk walking

Climbing stairs

Carrying a small load of shopping for 50 metres

Carrying or playing with a child

Gardening and housework

The end goal of the VILPA program is to provide new opportunities for people transitioning to retirement to accumulate sufficient physical activity to meet the World Health Organization guidelines.

Please review the following to provide medical clearance for your client if deemed suitable.

My client has no conditions that prevent them from increasing their physical activity.

My client’s heart condition is suitable to do VILPA.

My client’s blood pressure condition is suitable to do VILPA.

My client does not have known experience of chest pain when doing physical activity.

My client’s balance is suitable for increasing their physical activity.

My client’s bone & joint conditions are suitable for increasing their physical activity.

My client does not have any other conditions that prevent them from doing VILPA.

**Appendix 3: VILPA intervention booklet**

Vigorous Intermittent Lifestyle Physical Activity booklet

for

adults transitioning to retirement

**Content***

VILPA information

Pre-program measurements

My Goals

VILPA Checklist Instruction

VILPA checklists Week 1 to 6

Mid-way program measurements

My Goals – Revised

VILPA checklists Week 6 to 12

End-of-program measurements

Research team contacts

*NOTE: The original intervention booklet includes 12 VILPA checklists, one for each week. The format of the checklists is the same. For the purpose of this supplementary material, we included one checklist. The original booklet also included a map of the measurement location and contacts of two research team members. We removed this information in this supplementary material.

**VILPA information**

The World Health Organization (WHO) recommends adults accumulate minimum 75 minutes of high intensity physical activity or 150 minutes of moderate intensity physical activity each week. People who meet these physical activity recommendations have a lower risk of getting some cancers, heart disease, diabetes, and dementia. More than half of Australian adults do not meet WHO’s recommendation. This project aims to test an intervention to help people transitioning to retirement accumulate physical activity.

Existing programs to promote physical activity in adults tend to focus on exercises at the gym, participation in sports, or walking for 30 to 60 minutes each day. There are many obstacles to doing these exercise programs.

In this project, we are testing a Vigorous Intermittent Lifestyle Physical Activity (VILPA) program. The VILPA program does not require you to have special exercise skills.

The VILPA program was developed by reviewing international literature and co-designed with people transitioning to retirement and health professionals. The program contains activities that can be done anywhere and anytime. The program has the following features:

- The physical activity in this program will be at high intensity, the person doing the activity would have increased breathing rate, and might be ‘huffing and puffing’.
- The activities will only last a few minutes each time.
- The activities involve in the intervention will be daily activities that already exist in your life such as
  - Brisk walking
  - Climbing stairs
  - Carrying a small load of shopping
  - Carrying / Playing with children
- You will not require to have special training or any equipment to perform the activities.

**Benefits of VILPA**

A study on VILPA in 2022 reported that people who engage in VILPA three times a day (lasting 1 or 2 minutes each time) showed a 38 – 40 % reduction in all-cause and cancer mortality risk and a 48% reduction in cardiovascular disease mortality risk.

**Pre-program measurements**

Measurements will be conducted at Curtin University.

What to bring to my pre-program measurements:

Comfortable and supportive shoes to walk in

Comfortable clothing to walk in

Water bottle

Medical clearance letter signed by my GP

**My measurement records**

| Resting Heart Rate (Beats per minute) |  |
| --- | --- |
| Resting Blood Pressure (Systolic over diastolic) |  |
| Six-Minute Walking Test Distance (Metres) |  |
| My vigorous heart rate (Beats per minute) |  |

**My GOALS**

| 1 |  |
| --- | --- |
| 2 |  |
| 3 |  |
| 4 |  |
| 5 |  |

**My WhatsApp Group**

|  |
| --- |

**VILPA Checklist Instruction**

There are 12 VILPA weekly checklists in this booklet, one for each week.

At the beginning of each week, please review your plan for the week and identify potential daily activities that could reach a vigorous level.

Write down a few ideas or options of VILPA for each day in the top third of the checklist.

There are examples of VILPA in the mid-section of the checklist.

Once you completed any of the self-identified VILPA or the example VILPA, tick the box next to the activity, and add the total minute of the activity you completed.

If you did not complete VILPA each day, please note down reasons why you could not complete it.

If you have any questions about the checklists, please contact the research team.

| **WEEK # Possible VILPA** | | | | | | |
| --- | --- | --- | --- | --- | --- | --- |
| **Monday** | **Tuesday** | **Wednesday** | **Thursday** | **Friday** | **Saturday** | **Sunday** |
| **Activities completed** | | | | | | |
| Climb stairs  Carry shopping for 50metres+  Carrying or playing with child/ren  Cycling  Gardening  House cleaning  Pushing or moving heavy objects  Vacuuming  Walking uphill  Walking briskly  Walking to shops  Others  ………………………. | Climb stairs  Carry shopping for 50metres+  Carrying or playing with child/ren  Cycling  Gardening  House cleaning  Pushing or moving heavy objects  Vacuuming  Walking uphill  Walking briskly  Walking to shops  Others  ………………………. | Climb stairs  Carry shopping for 50metres+  Carrying or playing with child/ren  Cycling  Gardening  House cleaning  Pushing or moving heavy objects  Vacuuming  Walking uphill  Walking briskly  Walking to shops  Others  ………………………. | Climb stairs  Carry shopping for 50metres+  Carrying or playing with child/ren  Cycling  Gardening  House cleaning  Pushing or moving heavy objects  Vacuuming  Walking uphill  Walking briskly  Walking to shops  Others  ………………………. | Climb stairs  Carry shopping for 50metres+  Carrying or playing with child/ren  Cycling  Gardening  House cleaning  Pushing or moving heavy objects  Vacuuming  Walking uphill  Walking briskly  Walking to shops  Others  ………………………. | Climb stairs  Carry shopping for 50metres+  Carrying or playing with child/ren  Cycling  Gardening  House cleaning  Pushing or moving heavy objects  Vacuuming  Walking uphill  Walking briskly  Walking to shops  Others  ………………………. | Climb stairs  Carry shopping for 50metres+  Carrying or playing with child/ren  Cycling  Gardening  House cleaning  Pushing or moving heavy objects  Vacuuming  Walking uphill  Walking briskly  Walking to shops  Others  ………………………. |
| **Why I did not complete my goals…** | | | | | | |
| Feeling unwell*  ………………………. | ………………………. | ………………………. | ………………………. | ………………………. | ………………………. | ………………………. |

**Mid-way program measurements**

Measurements will be conducted at Curtin University.

What to bring to my pre-program measurements:

Comfortable and supportive shoes to walk in

Comfortable clothing to walk in

Water bottle

**My VILPA checklists from Week 1 to 6**

**My mid-way measurement records**

| Resting Heart Rate (Beats per minute) |  |
| --- | --- |
| Resting Blood Pressure (Systolic over diastolic) |  |
| Six-Minute Walking Test Distance (Metres) |  |
| My vigorous heart rate (Beats per minute) |  |

**My GOALS**

| 1 |  |
| --- | --- |
| 2 |  |
| 3 |  |
| 4 |  |
| 5 |  |

**My WhatsApp Group**

|  |
| --- |

**End-of-program measurements**

Measurements will be conducted at Curtin University.

Map:

What to bring to my pre-program measurements:

Comfortable and supportive shoes to walk in

Comfortable clothing to walk in

Water bottle

**My VILPA checklists from Week 6 to 12**

**My end-of-program measurement records**

| Resting Heart Rate (Beats per minute) |  |
| --- | --- |
| Resting Blood Pressure (Systolic over diastolic) |  |
| Six-Minute Walking Test Distance (Metres) |  |
| My vigorous heart rate (Beats per minute) |  |

**Research Team Contacts**

Contacts

**Appendix 4: Data collection and health outcome measures**

Sociodemographic information was collected upon each participant’s enrolment into the study; this included age, gender, highest level of education, self-reported number of chronic health conditions, manual or non-manual job type before retirement, postcode of residence, date of retirement or date of intention to retire.

Health outcome measures included the following:

- General health and wellbeing were measured by SF-36. Changes in self-reported general health in relation to PA were determined by scores in the SF-36 categories of physical functioning, physical role limitations, physical role limitations and general health. Wellbeing changes in the study were determined by scores in the SF-36 categories of social functioning, emotional role limitations and mental health.
- Heart rate and blood pressure were measured using an automated blood pressure monitor (Omron HEM7121). To identify the vigorous-intensity heart rates of participants in the intervention group, heart rate and blood pressure were also measured once immediately after their six-minute walk test (6MWT).
- Functional Fitness was assessed with the 6MWT. The total distance achieved in six minutes was recorded.

**Appendix 5: Summary findings of self-reported habit formation and the situational motivational scale at the end of the 12-week intervention.**

| Self-reported habit formation | | Mean (SD) |
| --- | --- | --- |
| No. | Contents | End of trial |
| **VILPA is something…** | | |
| 1 | I do frequently. | 4.35 (0.70) |
| 2 | I do automatically. | 3.94 (0.83) |
| 3 | I do without having to consciously remember. | 3.41 (1.18) |
| 4 | that makes me feel weird if I do not do it. | 3.41 (1.00) |
| 5 | I do without thinking. | 3.71 (1.05) |
| 6 | that would require effort not to do it. | 3.29 (1.16) |
| 7 | that belongs to my (daily, weekly, monthly) routine. | 4.24 (0.56) |
| 8 | I start doing before I realise I am doing it. | 3.65 (0.93) |
| 9 | I would find hard not to do | 3.29 (0.99) |
| 10 | I have no need to think about doing. | 3.29 (0.99) |
| 11 | That's typically "me". | 3.53 (0.87) |
| 12 | I have been doing for a long time. | 3.18 (1.01) |
| The situational motivational scale | | Mean (SD) |
| No. | Contents | End of trial |
| **Why are you currently engaging in VILPA?** | | |
| 1 | Because I think that this activity is interesting. | 5.94 (1.09) |
| 2 | Because I am doing it for my own good. | 6.71 (0.59) |
| 3 | Because I am supposed to do it. | 3.41 (1.91) |
| 4 | There may be good reasons to do this activity, but personally, I don't see any. | 1.35 (1.00) |
| 5 | Because I think that this activity is pleasant. | 5.12 (1.50) |
| 6 | Because I think this activity is good for me. | 6.12 (1.65) |
| 7 | Because it is something that I have to do. | 2.94 (1.85) |
| 8 | I do this activity but I am not sure if it is worth it. | 1.47 (1.01) |
| 9 | Because this activity is fun. | 5.12 (1.36) |
| 10 | By personal decision. | 5.94 (2.01) |
| 11 | Because I don't have any choice. | 1.18 (0.39) |
| 12 | I don't know; I don't see what the activity brings to me. | 1.81 (1.52) |
| 13 | Because I feel good when doing this activity. | 6.29 (0.92) |
| 14 | Because I believe this activity is important to me. | 6.41 (0.94) |
| 15 | Because I feel that I have to do it. | 2.65 (2.12) |
| 16 | I do this activity, but I am not sure it is a good thing to pursue it. | 1.12 (0.33) |

At the end of the intervention, participants reported engaging in different types of VILPA-like activities, walking briskly daily (n=12), gardening weekly (n=10), and pushing/moving heavy objects sometimes (n=10). Participants also reported engaging in dancing (n=3), jogging (n=3), and other forms of structured exercises (swimming (n=1), yoga (n=1) and Pilates (n=1)).

NOTE: For the self-reported habit formation, the Likert scale for each item was converted to a score: 1 = strongly disagree, 2 = disagree, 3 = neither agree or disagree, 4 = agree, 5 = strongly agree. For the situational motivational scale, the Likert scale for each item was converted to a score: 1 = does not correspond at all, 2 = corresponds very little, 3 = corresponds a little, 4 = corresponds moderately, 5 = corresponds a lot, 6 = corresponds mostly, 7 = corresponds exactly.
